# Management of complications after laparoscopic Nissen's fundoplication: a surgeon's perspective

**DOI:** 10.1186/1750-1164-3-1

**Published:** 2009-02-04

**Authors:** Tarun Singhal, Santosh Balakrishnan, Abdulzahra Hussain, Starlene Grandy-Smith, Andrew Paix, Shamsi El-Hasani

**Affiliations:** 1 The Princess Royal University Hospital, Bromley Hospitals NHS Trust, Farnborough Common, Orpington, Greater London, Kent. BR6 8ND. UK

## Abstract

**Introduction:**

Gastro-oesophageal reflux disease (GORD) is a common problem in the Western countries, and the interest in the minimal access surgical approaches to treat GORD is increasing. In this study, we would like to discuss the presentations and management of complications we encountered after Laparoscopic Nissen's fundoplication in our District General NHS Hospital. The aim is to recognise these complications at the earliest stage for effective management to minimise the morbidity and mortality.

**Methods:**

301 patients underwent laparoscopic treatment for GORD by a single consultant surgeon in our NHS Trust from September 1999. The data was prospectively collected and entered into a database. The data was retrospectively analysed for presentations for complications and their management.

**Results:**

Surgery was completed laparoscopically in all patients, except in five, where the operation was technically difficult due to pre-existing conditions. The complications we encountered during surgery and follow-up period were major intra-operative bleeding (n = 1, 0.33%), severe post-operative nausea and vomiting (n = 1, 0.33%), wound infection (n = 3, 1%), port-site herniation (n = 1, 0.33%), wrap-migration (n = 2, 0.66%), wrap-ischaemia (n = 1, 0.33%), recurrent regurgitation (n = 4, 1.32%), recurrent heartburn (n = 29, 9.63%), tension pneumothorax (n = 2, 0.66%), surgical emphysema (n = 8, 2.66%), and port-site pain (n = 4, 1.33%).

**Conclusion:**

Minimal access approach to treat GORD has presented with some specific and unique complications. It is important to recognise these complications at the earliest possible stage as some of these patients may present in an acute setting requiring emergency surgery. All members of the department, and not just the members of the specialised team, should be aware about these complications to minimise the morbidity and mortality.

## Introduction

Gastro-oesophageal reflux disease (GORD) is a common problem in the western world with an incidence of 5 per 1000 population per year [1]. The majority of patients have mild symptoms requiring only occasional treatment. However, a minority have persistent symptoms refractory to medical management and may go on to develop significant complications. This latter group initiated the interest in minimal access surgical approaches to treatment pioneered in the early 1990s.

Studies have confirmed the strong association between GORD and the risk of oesophageal adenocarcinoma [2]. GORD is also implicated in the aetiology of a number of related disorders [3]. This has sparked a resurgence of interest in methods not merely of symptom control, but of reflux prevention and cure. The proven safety and efficacy of Laparoscopic Anti-Reflux Surgery (LARS) has increased acceptance and demand for this treatment among patients and healthcare providers [4-9].

We have been offering LARS as a modality of treatment for GORD in our district general hospital (DGH) since September 1999. We report on the incidence and presentations of complications arising from this surgery and propose ways of overcoming them.

## Methods

The 301 consecutive patients undergoing LARS by a single consultant surgeon from September 1999 were entered into a prospective database. Ethics committee approval was not required for this procedure. Patients referred to our team from primary or secondary care with symptoms suggestive of chronic GORD were evaluated by taking a detailed history of symptoms and risk factors. Most patients had received initial treatment with Proton pump inhibitors (PPI) and pro-kinetic agents in primary care [10]. They were given advice regarding avoidance of risk factors predisposing to GORD and underwent upper Gastro-Intestinal (GI) Endoscopy, pH and manometric studies before a final decision to perform LARS was made. All intraoperative and postoperative complications were entered into the database. Selection criteria for patients undergoing LARS in this study are outlined in Table 1. Postoperative clinic follow up for all patients was at 2 weeks, 3 months, and one year.

**Table 1:** Selection Criteria for Patients undergoing LARS (n = 301)

▪ Persistent symptoms of reflux for over a year plus 1 or more of the following:
- Symptoms of volume reflux
- Patients responding well to PPI, but unwilling to take pills indefinitely
- Symptoms incompletely controlled by medications
- Respiratory symptoms

### General anaesthetic technique

A single consultant anaesthetist anaesthetized all but 2 patients. This enabled refinement of the technique, particularly with reference to early detection and treatment of pneumothorax, appropriate pain management, and post operative nausea and vomiting (PONV) prophylaxis.

Patients were induced and maintained with intravenous (I.V.) Propofol and Remifentanil (Synthetic opioid). The use of a processed EEG monitor was helpful in confirming an adequate depth of anaesthesia.

Where PONV occurred, it was treated aggressively with anti-emetics, IV fluids and occasionally by the use of Dexamethasone. Post-operative analgesia consisted of regular mild analgesics; usually Paracetamol and Ibuprofen, supplemented by subcutaneous morphine in small doses.

### Surgical technique

A single consultant surgeon performed all procedures with a standard 5 port incision.

The fundus of the stomach was mobilised completely by dividing the Gastro-splenic omentum including the short gastric vessels usually commencing from the splenic hilum to the gastro-oesophageal junction (GOJ). Circumferential dissection of the GOJ was completed preserving both vagi. A rubber sling was used through the retro-oesophageal window to lift the GOJ; further peri-oesophageal dissection, extending into the lower mediastinum, if needed, was done to deliver at least 3 cm of oesophagus into the abdominal cavity without tension. Crural repair was performed with interrupted sutures of 1-0 Ethibond sutures, and care was taken to prevent narrowing of the hiatus. The needle was left in situ in the lower most hiatal stitch to facilitate wrap fixation later. The posterior wall of the fundus was then brought around the GOJ behind the lower end of the oesophagus.

The possibility of oesophageal rotation was ruled out by the free to and fro passage of the fundus behind the oesophagus (Shoe-shine test). The fundus was brought through the retro-oesophageal window and all traction was released to confirm absence of tension in the wrap (Drop test). One (1-0) Ethibond suture was placed on the posterior 1/3 of each of the crura and left long to facilitate wrap fixation. A 3 cm floppy 360° fundic wrap was now constructed around the lower end of the oesophagus by suturing the anterior and posterior fundic walls around the oesophagus with several interrupted full thickness sutures without including the oesophageal wall within the sutures. The length of the intra-abdominal oesophagus and wrap were accurately measured* intra-operatively. Three-point fixation of the wrap was now carried out by fixing the wrap inferiorly to the lowest stitch of the crural repair and superiorly to the right and left crux respectively using the sutures with the needles left in-situ. All the needles and sling were now withdrawn and haemostasis confirmed.

#### * Method of measurement

❑ Steristrip was applied to a straight 5 mm dissector on which accurate markings were made so that the first mark was 3 cm from the tip of the instrument.

❑ Trade-markings near the tip of the ultrasonic dissectors gave an approximation of the length of the wrap.

## Results

301 patients underwent Laparoscopic anti-reflux surgery at our centre. The average age of the patients was 46 years (Range: 18 – 77 years) with a median age of 47 (Mode 53). Male to female ratio was1.88: 1.

**Figure 1:**
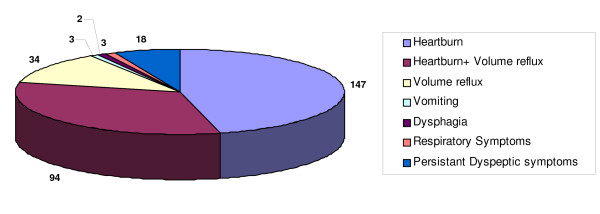
**Patient distribution by symptoms (n = 301)**.

The most common presenting symptoms were heartburn and volume reflux as illustrated in figure 1.

**Figure 2:**
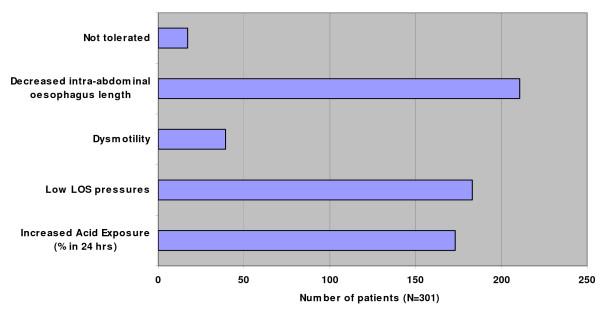
**Findings of pH and manometric study**.

The results of pH testing and manometry are depicted in figure 2.

Surgery was completed laparoscopically in all patients, except in five, where the operation was technically difficult due to pre-existing conditions (Table 2).

**Table 2:** Causes for inability to perform LARS in five patients (n = 301)

▪ Dense fibro-fatty peri-oesophageal and omental tissue
▪ Extreme omental and intra-abdominal adiposity
▪ Large, floppy liver making retraction difficult and risky
▪ Severe epigastric port site bleeding
▪ Dense fibrotic adhesions between stomach, liver and anterior abdominal wall following previous highly selective vagotomy

We had a good record of safety with no mortality and low morbidity. All complications, their presentations, and management were entered into a database (Table 3).

**Table 3:** Complications (n = 301)

Intraoperative bleeding	1	0.33%
PONV unresponsive to antiemetics	1	0.33%
Tension pneumothorax	2	0.66%
Surgical emphysema	8	2.66%
Postoperative wound infection	3	0.99%
Painful port-sites	4	1.33%
Postoperative laparoscopic port site hernia	1	0.33%
Postoperative dysphagia	11	3.65%
Wrap migration	2	0.66%
Wrap ischaemia	1	0.33%
Recurrent regurgitation	4	1.33%
Recurrent heartburn	29	9.63%

## Discussion

The cardinal principles of the operation are to restore the high-pressure zone and length of lower oesophagus exposed to the abdominal pressure, repair of the hiatal defect, and a short floppy fundoplication around the lower oesophagus [10,11]. This is achieved by hiatal and lower mediastinal oesophageal dissection [12], reduction of the hiatus hernia, and division of short gastric vessels to release the fundus from the spleen. We perform three-point fixation of the wrap to the crura to decrease the incidence of wrap migration and rotation. The oesophagus was not included in the sutures inserted to create the wrap in order to prevent oesophageal injury.

In most patients with large hiatus hernias, adequate intra-abdominal length can be achieved by a meticulous and higher mediastinal dissection [12]. We encountered failure in two cases where oesophageal shortening had to be treated by a Leigh Collis type gastroplasty. Accurate intraoperative measurements of the oesophageal intra-abdominal length after full mobilisation were made. A shortened oesophagus may predispose to transdiaphragmatic herniation of the wrap; adequate mobilisation of the oesophagus may prevent this complication in patients who have otherwise had a routine repair with uneventful postoperative recovery [12].

### Wrap Migration/Transdiaphragmatic Herniation

Wrap migration is synonymous with transdiaphragmatic herniation. These patients can present acutely with life threatening complications or may have longstanding herniation with chronic post-operative reflux or retro-sternal pain symptoms. Incidence of wrap migration ranges between 7% – 20% in published literature and accounts for up to 84% of failed laparoscopic repairs [13-16].

Only two patients (0.714%) had acute wrap migration or herniation in our series, and both presented in an acute setting. One of these patients was further complicated by ischaemic perforation of the strangulated gastric greater curvature. One patient had presented after ingestion of more than a litre of beer, though we routinely advise all our patients to avoid carbonated drinks for six weeks after surgery [16].

Some studies have demonstrated an increased incidence of wrap migration following laparoscopic repair of the hiatus rather than open repair. Reasons cited for this include: a tendency to extend the laparoscopic peri-oesophageal dissection high into the mediastinum, fewer adhesions and reduced postoperative pain allowing greater abdominal force to be transmitted to the hiatal region in the initial postoperative period [13]. However, the incidence of acute wrap migration in our series is low compared to others [13-16]. The strategies we employed to reduce this complication were mobilisation of the oesophagus to achieve adequate length of the intra-abdominal oesophagus, routine suture repair of the hiatus (posterior crural repair in particular), three point wrap fixation, good post-operative analgesia and anti-emetics, such as ondansetron [13,14]. Posterior crural repair was snug and made with non-absorbable material like Ethibond that can hold the repair for prolonged periods [14].

Longstanding wrap herniation or migration may present with symptoms of chronic postoperative reflux with or without dysphagia or odynophagia [14,15]. We investigated such patients with an OGD followed by CT-Scan or Barium meal. However, these investigations may not be accurate enough for small hernias thus the only solution in symptomatic patients is to dismantle the complete repair laparoscopically (small transdiaphragmatic hernias are almost like a Richter's Hernia and may become evident only on taking down the full wrap) and redoing it carefully [14,16]. In such cases laparoscopy will provide diagnostic as well as, therapeutic benefits.

### Wrap ischaemia with perforation

Wrap ischaemia is a distinct possibility in elderly patients with arteriopathy. In such cases, after mobilising the fundus, a partial wrap may be constructed instead of a full wrap which is likely to decrease interstitial resistance to blood flow.

Only one patient had wrap ischaemia with perforation, though that was compounded by wrap migration as described previously. This patient presented three months after a successful fundoplication with progressive epigastric pain and vomiting. The symptoms had started after consuming about two litres of beer. Urgent chest x-rays and CT scan showed wrap migration and gastric perforation. Laparotomy through a midline incision showed wrap migration and ischaemic perforation of the strangulated gastric greater curvature. Stapled partial gastrectomy was performed and GOJ was fixed to the crura. The patient remains symptom free for the last 16 months in spite of having no wrap.

### Postoperative dysphagia

No patients had absolute postoperative dysphagia in our series.11 patients reported dysphagia of varying degrees at 3 months post surgery. One patient had substantial weight loss at four months and underwent revision surgery.

8 patients had persistent dysphagia for solid food at nine months, but had not lost any weight. These patients were investigated with a Barium swallow and an upper GI Endoscopy. They were initially treated with gentle oesophageal dilatation.

Three patients who had a variable result with dilatation, but not complete resolution of dysphagia, elected not to have any further surgical procedure. The other five patients with dysphagia not responding to dilatation underwent a re-look operation with treatment of the cause. Two patients had hiatal stenosis due to fibrosis, which was released and the wrap was converted to partial posterior wrap with resolution of dysphagia; however, one patient developed minor reflux after the procedure. In the other three patients, no cause of dysphagia was found and the original wrap was completely dismantled and then converted to a partial posterior wrap, which relieved the symptoms of dysphagia.

In our experience, balloon dilatation could be tried within the first 3 months to manage early dysphagia; however, if it was not beneficial then there was no benefit in repeating it for significant dysphagia after 3 months.

Complete mobilisation of the gastric fundus with division of the short gastric vessels (Classic Nissen's fundoplication) ensures a floppy tension-free fundoplication, thus preventing dysphagia from a tight wrap or twisted oesophagus [11,17-19]. An incompletely mobilised fundus pulls tightly around the oesophagus, thereby twisting the lower oesophagus and the fundoplication valve itself, and leading to postoperative dysphagia. Many studies have failed to reveal any benefit from complete short gastric vessels division [20-22]. This remains an issue under debate.

Dysphagia due to hiatal stenosis following crural repair has been reported [15]. Various groups are currently analysing the benefits and safety of tension free prosthetic mesh repair over sutured repair of hiatus hernia with promising results [23-27]. However, we did not use the mesh to repair hiatus hernias at this stage. Needless to say, whenever these patients have required a revision procedure, due to dense adhesions with the prosthetic mesh, the revision surgery becomes extremely difficult [13,25]. The mesh may also erode into the oesophagus.

If after taking posterior crural stitches, the repair appears too tight, an anterior slit could be made on the arch of the crura to relive the constriction around the oesophagus. With a good three point anterior fixation, the wrap is unlikely to herniate anteriorly.

### Recurrent regurgitation

Four patients had recurrent volume reflux three months after surgery. Investigations suggested a shortened oesophagus. Two patients underwent revisional surgery with a Leigh-Collis type gastroplasty with anterior fundoplication stitched to the gastric tube. One patient had complete resolution of reflux. Fundoplication could not be completed in the second patient as he developed tension pneumothorax intra-operatively; his regurgitation has persisted. The third patient with a large hiatus hernia underwent distal gastrectomy with Roux-en-Y gastro-jejunostomy as an adequate length of intra-abdominal oesophagus could not be obtained. This cured his symptoms.

Complete mobilisation of the gastric fundus with floppy 360° wrap with an adequate length is a crucial step in LARS. We routinely divide the short gastric vessels to adequately mobilise the fundus. It has been widely reported that fundoplication operations dramatically reduce the number of transient lower oesophageal sphincter relaxations occurring both in the basal states as well as after artificial gastric distension or meal ingestion. It is accepted that this effect is essential to prevent further reflux.

The static mechanical effects of a total fundoplication are the same irrespective of whether all short gastric vessels have been divided or not. However, Engström et al have reported significantly more transient lower oesophageal sphincter relaxations in patients with intact short gastric vessels after a laparoscopic total fundoplication [28]. They believe that this could be explained by the unavoidable partial denervation of the proximal stomach area without completely dividing short gastric vessels. This may affect the triggering of mechano-receptors in the proximal part of the stomach, giving rise to transient lower oesophageal sphincter relaxations [28].

### Recurrent heartburn

Twenty-nine patients suffered recurrent heartburn after surgery. The symptoms were diminished compared to preoperative state, but they continued to need regular or intermittent treatment with PPI. All of these patients appeared to have a small volume wrap when visualised by retroflexion manoeuvre at gastroscopy. All patients were followed up. Six patients had revision surgery.

One patient had a short intra-abdominal oesophagus. An oesophageal lengthening procedure was performed with an anterior wrap, and this was successful in relieving the heartburn at 3 years after surgery.

In five patients, a longer (5 cm) wrap was fashioned after completely dismantling the previous wrap. This was successful in relieving the heartburn in four of them.

We had six patients with mild recurrent symptoms requiring intermittent PPI. No definitive cause for recurrent symptoms could be found in these patients as post-operative OGD and oesophageal pH readings were normal. These patients did not wish to have any further surgery.

Twelve patients were satisfied that their symptoms had improved, did not want any further investigations, and continued with intermittent PPI. Two patients are awaiting revision surgery. Three patients were lost to follow up.

Recurrent symptomatic heartburn continues to be an issue evading full explanation. There is an emerging belief that there need not be a correlation between position and integrity of the wrap and the results of the surgery [29]. Various authors have seen definitive benefit in revision surgery for recurrent symptoms resulting from anatomical and functional failures recognised post-operatively [30]. Attempting a fundoplication without achieving an adequate intra-abdominal length of oesophagus could be a frequent cause of surgical failure. Actual intra-operative measurement of intra-abdominal oesophageal and wrap length, as we did in our series, may help to recognise inadequate intra-abdominal length of the oesophagus and/or length of the wrap [31,32].

The absence of significant reflux symptoms in our patients who had to have the fundoplication undone or resected post operatively due to intra-operative or post-operative wrap related problems highlights the importance of restoration of adequate intra-abdominal length of oesophagus to the functional end result of the operation [11,12].

Successful LARS leads to a significant improvement in quality of life of patients [4,33,34]. Only 16 patients in our series needed to use proton pump inhibitors post operatively for recurrent symptoms. None of them were on regular proton pump inhibitors. Even these patients feel that their quality of life has improved significantly since they don't need to take daily medications.

In terms of therapeutic advantage, LARS achieves symptomatic relief with abolition of mechanical reflux [4,35]. This protects the oesophageal mucosa from injury due to the non-acidic components of the refluxate [12,33,34,36]. This may have an added advantage in promoting mucosal healing, reversal of mucosal changes caused by long standing reflux disease and preventing sequelae of longstanding reflux like Barrett's disease and strictures [37]. This could provide the key to decreasing the incidence of pre-malignant change and possibly lower the incidence of oesophageal cancers, which have been increasing in prevalence over the last decade.

### Pneumothorax

Four patients had a small pneumothorax, and two patients had an inadvertent tension pneumothorax intraoperatively. Tension pneumothorax occurred on the left side in both patients during trans-hiatal dissection for revisional surgery.

In our experience, a small pneumothorax occurring towards the end of the procedure is of little consequence as the CO_2 _is absorbed very quickly from the pleural cavity [15]. However, a pneumothorax in the middle of the procedure may make the ventilation of the lungs difficult, and could also result in tension pneumothorax with collapse of the ipsilateral lung and compromising cardio-vascular system. Inserting an inter-costal drain intraoperatively would not be useful as the pneumoperitoneum would be lost through the drain.

Careful, millimetre-by-millimetre dissection and meticulous haemostasis to prevent any deterioration in view of the operative field during trans-hiatal peri-oesophageal dissection is necessary to minimise the risk of this complication [15]. However, should it inadvertently happen, we immediately lower the pneumoperitoneum to 8 mm of Hg, and try to plug the defect in the pleura with omentum or stomach. This strategy has been successfully used in our patients, and we completed all the procedures laparoscopically. We had tried to stitch the pleura intraoperatively in one patient, but did not find this to be an effective method.

### Surgical emphysema and pneumomediastinum

In our series, a degree of surgical emphysema was relatively common. Detection can be difficult as the early signs are subtle and non-specific.

It was usually first palpable in the left supra-clavicular area, then becoming bilateral and extending into the neck. Two patients had surgical emphysema involving the eyelids.

The earliest physiological signs were a gradually increasing heart rate, although this was not a specific sign, as we commonly noted tachycardia and hypertension after inducing pneumoperitoneum. It was more common in slim patients and those having more extensive or prolonged mediastinal dissections.

Surgical emphysema and pneumomediastinum are inadvertent but unavoidable complications of high mediastinal dissection to achieve adequate intra-abdominal length of oesophagus. All patients need to be forewarned about this complication, as it is often difficult to make accurate predictions preoperatively about the possibility of short oesophagus necessitating high mediastinal dissection. Even in extensive cases, patients usually need only reassurance [15].

Intra-operative detection of a developing pneumothorax is difficult without a high degree of suspicion as the signs are non-specific. The operator may be unaware of perforating the pleura, as often there are no visual clues; however occasionally, the diaphragm bulges into the peritoneal cavity, which may be the earliest sign of pneumothorax. The early signs of this complication were usually gradual tachycardia and small rises in ventilatory pressures. Tachycardia is not a reliable sign on its own as it occurs commonly (with hypertension) after inducing a pneumoperitoneum.

In all cases of tension pneumothorax in our series, it was left sided. Diagnosis was by clinical suspicion and confirmed by careful auscultation of the lung fields. It is important to differentiate this from endobronchial migration of the endotracheal tube, which may easily occur after inducing pneumoperitoneum, as the carina is pushed cephalad.

Treatment in all cases was successful deflating the pneumoperitoneum for a brief period until cardio-respiratory parameters normalised, then plugging the defect if one was identifiable, and completing the procedure with the lowest possible intraperitoneal pressures.

### Post operative wound problems

Morphine is not an effective treatment for acutely painful port sites, and higher doses may contribute to PONV; injection of local anaesthetics at port sites may be the best option. Three patients with minor wound infections were treated conservatively.

### Post-operative nausea and vomiting

All patients received PONV prophylaxis; where PONV occurred, it was treated aggressively with anti-emetics, IV fluids and occasionally by the use of Dexamethasone. One patient required the insertion of a nasogastric tube few hours postoperatively for persistent nausea and retching, which was not responding to anti-emetics.

## Conclusion

The experience from our series shows that LARS can be provided in a cost effective manner in a DGH NHS setting. This assumes significance considering the prevalence of GORD in the population. LARS can save the community the cost of long-term treatment with PPI and improves the quality of life of patients.

Growing interest in the pathophysiology of GORD has led to technical refinements, which have made LARS effective with very low morbidity. The operation can be performed with minimal pain and postoperative discomfort. This has resulted in growing acceptability of the procedure among the patients and healthcare providers alike. The growing application of LARS to treatment of GORD could have a positive impact on the incidence and prevalence of both direct and indirect sequelae of the disease in the community.

Though LARS can be performed with low morbidity, a high index of suspicion needs to be maintained to detect early postoperative complications. We have seen that favourable outcomes depend on the rapidity of response to the acute complications. Most complications, when detected early, can be rectified laparoscopically avoiding the need for a major laparotomy. There is a need therefore to alert surgeons from other specialities who participate in acute general surgical take to these issues.

## Competing interests

The authors declare that they have no competing interests.

## Authors' contributions

TS wrote the article, participated in the sequence alignment and drafted the manuscript, SB participated in the sequence alignment, formatted the pictures and performed language corrections, AH collected the data and investigation studies, participated in the article design and critically evaluated the article, SGS collected the data and investigation studies, participated in the article design and critically evaluated the article, AP participated in the article design and critically evaluated the article, SEH conceived the study, and participated in its design and coordination, and helped to draft the manuscript. All authors read and approved the final manuscript.

## References

[B1] Dent J, El-Serag HB, Wallander MA, Johansson S (2005). Epidemilogy of Gastro-oesophageal reflux disease: a systematic review. Gut.

[B2] Turcotte S, Duranceau A (2005). Gastroesophageal reflux and cancer. Thorac Surg Clin.

[B3] Malfertheiner P, Hallerback B (2005). Clinical Manifestations and complications of gastroesophageal reflux disease (GERD). Int J Clin Pract.

[B4] Mahon D, Rhodes M, Decadt B, Hindmarsh A, Lowndes R, Beckingham I, Koo B, Newcombe RG (2005). Randomised clinical trial of Laparoscopic Nissen fundoplication compared with proton-pump inhibitors for treatment of chronic gastro-oesophageal reflux. Br J Surg.

[B5] Kamolz T, Granderath FA, Schwieger UM, Pointer R (2005). Laparoscopic Nissen Fundoplication in patients with nonerosive reflux disease. Long-term quality – of – life assessment and surgical outcome. Surg Endosc.

[B6] Granderath FA, Kamolz T, Schweiger UM, Haas CF, Wykypiel H, Pointer R (2002). Long-term results of laparoscopic antireflux surgery. Surgical outcome and analysis of failure after 500 laparoscopic antireflux procedures. Surg Endosc.

[B7] Bailey ME, Garrett WV, Nisar A, Boyle NH, Slater GH (2003). Day-case laparoscopic Nissen fundoplication. Br J Surg.

[B8] Dallemagne B, Weerts J, Markiewicz S, Dewandre J-M, Wahlen C, Monami B, Jehaes C (2006). Clinical results of laparoscopic fundoplication at ten years after surgery. Surg Endosc.

[B9] Ray S (2003). Results of 310 consecutive patients undergoing laparoscopic Nissen fundoplication as hospital outpatients or at a freestanding surgery centre. Surg Endosc.

[B10] Wilkerson PM, Stratford J, Jones L, Sohanpal J, Booth MI, Dehn TCB (2005). A poor response to proton pump inhibition is not a contradiction for laparoscopic antireflux surgery for gastroesophageal reflux disease. Surg Endosc.

[B11] Lord RVN, Demeester TR, Morris PJ, Wood WC (2000). Reflux Disease and Hiatus Hernia.

[B12] O'Rourke RW, Khanjanchee YS, Urbach DR, Lee NN, Lockhart B, Hansen PD, Swanstrom LL (2003). Extended transmediastinal dissection: an alternative to gastroplasty for short oesophagus. Arch Surg.

[B13] Hunter JG, Smith D, Branum GD, Waring P, Trus TL, Cornwell M, Galloway K (1999). Laparoscopic Fundoplication Failures. Patterns of Failure and Response to Fundoplication Revision. Annals of Surgery.

[B14] O'Boyle CJ, Heer K, Smith A, Sedman PC, Brough WA, Royston CMS (2000). Iatrogenic thoracic migration of the stomach complicating laparoscopic Nissen fundoplication. Surg Endosc.

[B15] Granderath FA, Schweiger UM, Kamolz T, Pointner R (2005). Dysphagia after laparoscopic antireflux surgery: a problem of hiatal closure more than a problem of the wrap. Surg Endosc.

[B16] Watson DI, de Beaux AC (2001). Complications of laparoscopic antireflux surgery. Surg Endosc.

[B17] Davis RE, Awad ZT, Flilpi CJ (2004). Technical factors in the creation of a "floppy" Nissen fundoplication. Am J Surg.

[B18] Huntington TR, Danielson L (2001). Variation in fundic dimensions with respect to short gastric vessel division in laparoscopic fundoplication. Surg Endocs.

[B19] Richardson WS, Hunter JG (1999). Laparoscopic floppy Nissen fundoplication. Am J Surg.

[B20] Engstrom C, Blomqvist A, Dalenback J, Lonroth H, Ruth M, Lundell L (2004). Mechanical consequences of short gastric vessel division at the time of laparoscopic total fundoplication. J Gastrointest Surg.

[B21] Sato K, Awad ZT, Filipi CJ, Selima MA, Cummings JE, Fenton SJ, Hinder RA (2002). Causes of Long-term dysphagia after laparoscopic Nissen fundoplication. JSLS.

[B22] O'Boyle CJ, Watson DI, Jamieson GG, Myera JC, Game PA, Devitt PG (2002). Division of short gastric vessels at laparoscopic nissen fundoplication: a prospective double-blind randomised trial with a 5-year follow-up. Ann Surg.

[B23] Casaccia M, Torelli P, Panaro F, Cavaliere D, Saltalamacchai L, Troilo BM, Savelli A, Valente U (2005). Laparoscopic tension-free repair of large paraesophageal hiatal hernias with a composite A-shaped mesh: two-year follow-up. J Laparoendosc Adv Surg Tech.

[B24] Franzides CT, Madan AK, Carlson MA, Stavropaulos GP (2002). A Prospective, Randomised Trial of Laparoscopic Polytetrafluoroethylene (PTFE) patch repair vs Simple Cruroplasty for Large Hiatal Hernia. Arch Surg.

[B25] Johnson JM, Carbonell AM, Carmody BJ, Jamal MK, Maher JW, Kellum JM, DeMaria EJ (2006). Laparoscopic mesh hiatoplasty for paraesophageal hernias and fundoplications: A critical analysis of the available literature. Surg Endosc.

[B26] Muller-Stich BP, Holzinger F, Kapp T, Klaiber C (2006). Laparoscopic hiatal hernia repair: Long term outcome with the focus on the influence of mesh reinforcement. Surg Endosc.

[B27] Granderath FA, Carlson MA, Champion JK, Szold A, Basso N, Pointner R, Frantzides CT (2006). Prosthetic closure of the oesophageal hiatus in large hiatal hernia repair and laparoscopic antireflux surgery. Surg Endosc.

[B28] Engstrom C, Blomqvist A, Dalenback J, Lonroth H, Ruth M, Lundell L (2004). Mechanical consequences of short gastric vessel division at the time of laparoscopic total fundoplication. J Gastrointest Surg.

[B29] Scheffer RCH, Samsom M, Frakking TG, Smout AJ, Gooszen HG (2004). Long term effect of fundoplication on motility of the oesophagus and oesophagogastric junction. Br J Surg.

[B30] Dutta S, Bamehriz F, Boghossian T, Pottruff CG, Anvari M (2004). Outcome of laparoscopic redo fundoplication. Surg Endosc.

[B31] Awad ZT, Mittal SK, Roth TA, Anderson PI, Wilfley Jr (2001). WA, Filipi CJ. Esophageal shortening during the era of laparoscopic surgery. World J Surg.

[B32] Mattioli S, Lugaresi ML, Di Simone MP, D'Ovidio F, Pilotti V, Bassi F, Brusori S, Gavelli G (2004). The surgical treatment of the intrathoracic migration of the gastro-oesophageal junction and of short oesophagus in gastro-oesophageal reflux disease. Eur J Cardiothorac Surg.

[B33] Papasavas PK, Keenan RJ, Yeaney WW, Caushaj PF, Gagné DJ, Landreneau RJ (2003). Effectiveness of Laparoscopic fundoplication in relieving the symptoms of gastroesophageal reflux disease (GERD) and eliminating antireflux medical therapy. Surg Endosc.

[B34] Granderath FA, Kamolz T, Schweiger UM, Pointer R (2002). Quality of Life, Surgical Outcome and Patient Satisfaction Three years after Laparoscopic Nissen Fundoplication. World J Surg.

[B35] Straathof JWA, Ringers J, Masclee AAM (2001). Prospective study of the effects of laparoscopic Nissen fundoplication on reflux mechanisms. Br J Surg.

[B36] Theisen J, Peters JH, Fein M, Huges M, Hagen JA, Demeester SR, Demeester TR, Laird PW (2005). The mutagenic potential of duodenooesophageal reflux. Arch Surg.

[B37] Stylopoulos N, Rattner DW (2005). The History of Hiatal Hernia. From Bowditch to Laparoscopy. Ann Surg.

